# Coding of touch properties by three types of mechanosensory cells of the leech hirudo medicinalis

**DOI:** 10.1186/1471-2202-14-S1-P224

**Published:** 2013-07-08

**Authors:** Friederice Pirschel, Jutta Kretzberg

**Affiliations:** 1 Computational Neuroscience, University of Oldenburg, D-26111 Oldenburg, Germany

## 

Many animals use tactile perception to perceive their environment. Tactile stimulation can elicit accurate behavioral responses, e.g. adjustment of body posture to uneven ground. The medicinal leech produces an extremely precise behavior, the local bend reflex [1], in response to touch stimuli. Behaviorally, leeches discriminate touch locations which are only 9° (<1 mm) of the body circumference apart [2]. The local bend was shown to depend on stimulus location and intensity [3]. It is initiated by activation of mechanosensory cells and processed by one layer of interneurons, causing one layer of motorneurons to elicit muscle contraction and elongation. This small neuronal network is located in each of the individual ganglia. Hence, behavioral responses to sensory stimuli can be elicited even if one ganglion with associated body wall is dissected from the rest of the CNS. One ganglion contains three types of mechanosensory cells: 6 T ('touch'), 4 P ('pressure') and 4 N ('noxious') cells, whereby the receptive fields of adjacent T cells as well as P cells strongly overlap [4].

Thomson and Kristan investigated in their study [2] the encoding of stimulus location based on two P cells with overlapping receptive fields and analyzed the differences of their spike counts and latencies. They found that latency differences allow reliable discrimination of touch locations with a distance of 4°. In contrast, discrimination based on spike count differences requires a touch location distance of 13°.

In order to investigate how the three types of leech mechanosensory cells respond to tactile stimulation, we recorded intracellularly from pairs of these neurons while stimulating the skin mechanically. Tactile stimuli varied in intensity and location. Responses of cell pairs were analyzed by calculating the differences of latencies and of spike counts. Discrimination performance was evaluated for location distances and intensity differences based on a pair-wise classification.

We found:

1. All three types of mechanosensory cells respond to strongly overlapping intensity ranges (≥ 50 mN). Spike count and response latency of all cell types depend on touch intensity as well as location. These results suggest that N cells could be involved in the encoding of touch stimuli.

2. For the estimation of touch location, we found in agreement with Thomson and Kristan [2] that latency difference of both P cells leads to reliable classification of small touch location distances, when touch stimuli of higher intensities (e.g. 50 mN) are used. Locations of touch stimuli of lower intensities (e.g. 10 mN) can better be discriminated based on latency difference of two T cells. Combinations of T and P cell responses do not improve discrimination.

3. Stimulus intensities are optimally discriminated by spike counts of single P cells. Relative response features do not improve the estimation of intensity, neither for pairs of the same type nor for different cell types.
